# Comparison of GnRh Agonist Microdose Flare Up and GnRh Antagonist/Letrozole in Treatment of Poor Responder Patients in Intra Cytoplaspic Sperm Injection: Randomized Clinical Trial

**DOI:** 10.5539/gjhs.v8n4p166

**Published:** 2015-08-06

**Authors:** Azar Nabati, Sepideh Peivandi, Alireza Khalilian, Sina Mirzaeirad, Seyyed Abbas Hashemi

**Affiliations:** 1 Department of Gynecology and Obstetrics, Mazandaran University of Medical Sciences, Sari, Iran; 2 Department of Community Medicine, Mazandaran University of Medical Sciences, Sari, Iran; 3 Department of Gynecology and Obstetrics, Immunogenetic Research Center, Mazandaran University of Medical Sciences, Sari, Iran

**Keywords:** GnRh agonist, letrozole, injection

## Abstract

**Backgrounds:**

the prevalence of infertility is up to 10 to 15 % which 9 to 24 % of them are Poor Ovarian Responders (POR). This study was designed to compare two methods of GnRH Agonist Microdose Flareup (MF) and GnRH Antagonist/Letrozole (AL) in treatment of these patients.

**Methods and Materials:**

this randomized clinical trial study consisted of 123 patients. In the first step of treatment in both methods FSH, LH, estradiol, anderostandion, testestron in third day of menstruation period and the thickness of endometrium by Transvaginal sonography were evaluated. At the time of HCG injection the thickness of endometrium and follicles which were more than 14mm ware established and hormones were evaluated. Two weeks later serum βhCG and after 6 to 8 weeks Transvaginal sonography were applied to prove the pregnancy.

**Results:**

there were 61 patients with mean age of 38.7±4.58 in MF group and 62 patients with mean age of 38.5±4.6 in AL group (P=0.80). At the time of hCG injection there were significant increase in the level of LH, estradiol, thickness of endometrium and follicles more than 14mm in MF patients (P<0.0001). The mean time of ovary stimulation in MF group was 10.72±1.5 and in AL was 8.45±1.2 (P<0.0001). The mean level of gonadotropin which were used was 80.6±20.1 in MF patients and 64.7±16.4 in AL group (P<0.0001). 18 % of MF group and 38.7% in AL group had no normal cycle of ovulation (OR: 2.87, 95% CI: 1.25-6.57, P=0.011). The mean numbers of oocyte and normal fetus in MF was 5.83±3.5 and 3.7±2.5 and in AL was 3±1.69 and 1.4±1.33 (P<0.0001). The number of chemical pregnancy in MF group was 10 (16.4%) and in AL was 3 (4.8%) (OR 3.85, 95% CI: 1.06-14.77, P=0.037). Clinical pregnancy in 10 patients (16.4%) of MF group and 3 (4.8%)in AL was reported. OR 3.85, 95% CI: 1.06-14.77, P=0.037).

**Conclusion:**

this study showed that MF method of pregnancy leads to more positive results in pregnancy based on chemical and clinical evaluation in comparison with AL and is advised for poor responder patients.

## 1. Introduction

Infertility is defined as emergence of pregnancy after one year of intercourse without contraception. 10-15% of couples in fertility ages experience infertility (Mosher et al., 1991). Invention of IVF methods and other ART technologies have caused major changes in treating infertility. About 9-24% of women treated by IVF are regarded as poor responders to ovarian stimulation (Mosher et al., 1991). In most cases, these individuals do not have appropriate respond to standard ovarian stimulation method and treatment cycles fail due to insufficient production of oocyte. This issue is one of the major problems in treating infertility (Davar et al., 2010), (Fasouliotis et al., 2000). Detection of these individuals is conducted based on age, clinical and laboratory investigation including number of follicles less than four pieces and the Estradiol level less than 1500 pg/m per day, injection of hCG (Benadiva et al., 1988) and increase in base level of FSH at the third day of period, reduction in Inhibine B level, small size of ovary due to reduction in number of ovary follicles and high dose of Gonadotropin required in IVF cycles (Tarlatzis et al., 2003). Today, by taking into account the increase in marriage age and late actions for pregnancy, the number of these patients is increasing. Thus, the way of treating these patients has been discussed and considered by centers for treating infertility (Tarlatzis et al., 2003; Yarali et al., 2009). Today, two protocols of GnRH Agonist Microdose Flareup (MF) and GnRH Antagonist/Letrozole (AL) are considered by researchers in treatment of poor responders due to shorter treatment duration and consumption of less Gonadotropin (Yarali et al., 2009; Sunkara et al., 2007; Kim et al., 2011). From one side, hormonal manipulation in order to increase the number of ovary follicles during the ovarian stimulation period and adding these treatments to the main protocols and improvement of results have provided the field for new researches regarding poor responders, including use of GH, complements containing LH, DHEA, Testosterone and Estradiol before commencement of protocols and Aromatase inhibitors during ovarian stimulation periods (Yarali et al., 2009).

Letrozole is a special non-steroidal Aromatase inhibitor which was first used in breast cancer in mono-pose women for suppression of estrogen products and today it is applied as the first line of induction in PCO women (Polycystic ovary) (Yarali et al., 2009). It seems that Aromatase inhibitors (Letrozole) cause reduction in the need for Gonadotropins during IVF (in vitro fertilization) and ICSI (intra cytoplasmic sperm injection) cycles by reduction in estrogen that this issue causes reduction in patient costs besides reducing Gonadotropins’ side effects (Mitwally et al., 2005). Mitwally et al. indicated that Aromatase inhibitors cause improvement in ovary’s respond to FSH in poor responder patients (Goswami et al., 2004). Thus, this study was defined to compare two protocols of GnRH Agonist Microdose Flareup and GnRH Antagonist/Letrozole in treating poor responder individuals referring to infertility center of Imam Khomeini hospital at Sari.

## 2. Materials and Methods

this study is a clinical trial which was conducted on 123 poor responder (imam khomeini hospital, sari, Iran) patients based on existing indicators which included the ones whose third day FSH was 110 to 15 mIU/m or their Estradiol level was < 1500 pg/m or the number of their mature follicles in previous IVF cycle was 3 pieces or less or their age was over 40 years old. The criteria for exiting the study: the individuals having had ovary surgery history of endometriosis, sustained hyperprolactinemia or other hormonal disorders, having single ovary, FSH more than 115 mIU/m or the ones whose husbands were Azoospermia were omitted from the study. Then, in case of having the condition for entering the study, they were entered. Besides that at the beginning of the treatment in both FSH and LH protocols the Estradiol, Androstenedione and testosterone were measured by Elecsys method at the third day of period at central laboratory of Sari and the elevation of endometrial and its thickness were investigated and recorded by Ttranse Vaginal Sonography (TVS). Then, one of the treatment methods started for them randomly. In group GnRH Agonist Microdose Flareup, 3 days after the end of OCP period, TVS was conducted and in case they had ovary’s suppression symptoms (endometrial thickness>5mm and follicles>10mm) the GnRH-agonist (Superfact; Aventis pharma, Frankfurt, Germany) with dose of 40 microgram per day was started subcutaneously and it continued by the day for prescription of hCG. Two days after commencement of GnRH-agonist, the F gonal ampoule (Gonal F; serono, Aubonne, Switzerland) with the dose of 6 pieces per day started and at the 8^th^ day of cycle, Sonography was conducted for investigating the follicular growth and regulating the dose of Gonadotropin. Then, the patients were proceeded by TVS. When at least three follicles ≤ 17 mm or more were found, hCG (Pregnyl; Organon, Oss, Netherlands) with the dose of 10000 units was injected and 36 to 38 hours later the oocytes were aspired from ovaries under conduction of Sonography and under anesthesia. The day of injection of hCG, the number of follicles higher than 14 mm and the endometrial thickness were recorded and the measurement for Estradiol level, LH, testosterone and Androstenedione was conducted. In group GnRH Antagonist/Letrozole, after completion of one period for consumption of OCP, Letrozole pill (Femara; Novartis, East Hanover, USA) was started from the third day of cycle with dose mg/d 5/2 and it continued for 5 days. Simultaneously from the third day of cycle, F Gonal with dose 450 IU was prescribed (Yarali et al., 2009) and at the 8^th^ day of cycle investigation of follicular growth was conducted via TVS. When at least 3 precursor follicles ≤17 mm was observed in TVS, HCG by 10000 units was prescribed and 34 to 38 hours later, oocytes were aspired from ovaries under conduction of Sonography and under anesthesia. The day for injection of hCG, the number of follicles higher than 14 mm and thickness of endometrial was recorded and again LH, Estradiol, Androstenedione and testosterone were measured by Elecsys method at central laboratory of Sari. In both two groups, from the day after weighing the oocytes, the progesterone was prescribed as inter-vaginal by the dose of 400 milligram twice a day. The number of oocyte and also the number of fetus gained and their quality in each patient was recorded. The day after puncture, maximum 3 fetuses were transmitted to the uterus.

Two weeks after transmission of fetus, hCG-β was conducted for investigation of chemically pregnancy and Sonography TVS 6 was conducted by 8 weeks later for investigation of clinical pregnancy. At the time of performing the research, the physician conducting Sonography was unaware of the ovary’s suppression method and prescription of Gonadotropin started for everyone with the same dose by the physician performing the Sonography and changes of dose was conducted depending to the rate of ovary’s respond. Prescription of medicine for ovary suppression was performed by IVF experts cooperating in the plan. The embryologist of the current study was also unaware of the treatment method. For data analysis, the SPSS software version 14 was applied.

The statistical tests were conducted for comparing quantitative and qualitative variables in two groups with t-test statistical method and χ^2^ test. p<0/05 was defined as significant.

### 2.1 Ethics

All subjects gave their written consent to participate in the study. This study was conducted in accordance with the Declaration of Helsinki and good clinical practice according to International Conference on Harmonisation guidelines. The ethics committee of Mazandaran Universtiy of Medical Sciences, Sari, IRAN approved this study (IRCT2014050516858N3).

## 3. Results

the number of patients present in the study was 123 persons that 61 patients with the age mean of 40.86±3.07 years (between 37 to 43 years old) in MF group and 62 patients with the age mean of 40.75±3.23 years (between 37 to 43 years old) in AL group were present. There were no significant statistical difference between the age of two groups (P=0.84). The primary data of patients are brought in table No. 1. As it is observed in this table, in comparing two groups on height, weight, BMI, infertility duration, the number of follicles at the beginning of induction and endometrial thickness at the beginning of induction no significant statistical difference was observed (P>0.05). Also, between FSH, LH, Estradiol, testosterone and Androstenedione on the third day of period, there were no significant statistical difference (P>0.05) (Table 1).

**Table 1:** Demographic data of study population

	AL	MF	P Value
Age(yr)	58.38±60.4	38.78±4.58	0.80
Height(meter)	1.56±0.04	1.57±0.05	0.59
Weight(kg)	67.67±6.94	67.62±7.00	0.96
BMI(kg/m^2^)	27.25±2.51	27.11±2.72	0.75
Infertility(yrs)	8.14±4.83	9.29±6.20	0.27
Number of follicle at time of induction	4.06±1.61	4.39±1.76	0.28
Endometrium thickness at time of induction	3.96±1.55	4.14±1.49	0.51
FSH level in third day of menstruation	11.06±2.47	11.13±2.24	0.87
LH level in third day of menstruation	4.82±1.77	4.65±1.82	0.60
estradiol level in third day of menstruation	65.84±37.03	64.46±40.16	0.84
testosterone level in third day of menstruation	5.67±1.57	4.11±2.31	0.33
androstandione level in third day of menstruation	1.78±0.90	1.77±0.90	0.95

In MF group, the level of FSH in 309 patients (63.9%) was less than 10 and in 22 patients (36.1%) was between 10 to 15. In AL group, 36 patients (58.1%) had FSH under 10 and 26 patients (41.9%) had FSH between 10 to 15. There were no significant statistical difference between the two group (P=0.50). In investigation of the background of cancelled cycle at the beginning of study, 17 patients in MF group (27.9%) and 18 patients in AL group (29%) had cancelled cycles that there were no significant statistical difference (P=0.88). investigation of infertility reason in two groups indicated that the reason of infertility in group MF in 18 patients was tubal (29.5%) and at 43 patients was idiopathic (73.5%). In group AL, the infertility reason for 20 patients (32.3%) was tubal and in 42 patients (67.7%) was idiopathic. there were no significant statistical difference (P=0.74). the patients’ data at the day of hCG injection was brought in Table 2. As it is observed in this table, there were no significant statistical difference between testosterone and Androstenedione in two groups (P>0.05). Meanwhile, the LH and Estradiol level and the number of follicles over 14 mm and endometrial thickness at the time of hCG injection were significantly higher in MF group (P<0.0001).

**Table 2:** Comparison of examined factor on the day of HCG injection

	MF	AL	P Value
Level of LH	49.86±18.02	24.7±6.6	0.000
Level of estradiol	1393.61±659.27	710.85±334.95	0.000
Level of testosterone	4.23±2.24	4.13±2.24	0.80
Level of androstandione	1.64±0.82	1.63±0.86	0.94
Number of follicles	8.41±3.27	90.4±1.90	0.000
Endometrium thickness	9.54±1.36	8.06±0.96	0.000

The mean for ovarian simulation period in MF group was 10.72±1.59 days and in AL group was 8.45±1.27 days that a significant statistical difference was observed in two groups (P<0.0001). The rate of consumption of Gonadotropin in groups MF and AL was 80.67±20.19 and 64.77±16.49. The rate of consumption of Gonadotropin in group MF was significantly higher (P<0.0001) (Table 3). In investigating the cancelled cycle after treatment, 11 patients in MF group (18%) and 24 patients in AL group (38.7%) had cancelled cycles. The number of cancelled cycles was significantly less in MF group and the chance of cancellation of cycles in group AL was 2.87 times more than group MF (OR: 2.87, 95% CI: 1.25-6.57, P=0.011) (Table 3). The mean of number of oocyte and number of proper fetus in group MF was 5.83±3.53 and 3.7±2.50, while these figures for group AL were 3.00±1.69 and 1.40±1.33. More statistical analysis indicated that the number of oocyte and number of proper fetus in group MF were significantly higher (P<0.0001) (Table 3). The number of chemical pregnancies in group MF was 10 cases (16.4%) and in group AL was 3 cases (4.8%). there was a significant statistical difference between the two group and the chance for chemical pregnancy in method MF was 3.85 times more than AL method (OR: 3.85, 95% CI: 1.06-14.77, P=0.037). besides that in this study, the cases of clinical pregnancy like the chemical pregnancy were positive in 10 patients of group MF (16.4%) and 3 cases in group AL(4.8%). Here again there was a significant statistical difference between the two group and the chance for chemical pregnancy in method MF was 3.85 times more than AL method (OR: 3.85, 95% CI: 1.06-14.77, P=0.037) (Table 3).

**Table 3:** Comparison of GnRh agonist microdose flare up and GnRh antagonist/letrozole in treatment of poor responder patients in intra cytoplaspic sperm injection

	MF	AL	P Value
Time of ovary stimulation	10.72±1.59	8.45±1.27	0.000
Mean usage of gonadotropin	80.67±20.19	64.77±16.49	0.000
Mean number of canceled cycles	**11 (18%)**	**24 (38.7%)**	0.011
Number of oocytes	5.83±3.53	3.00±1.69	0.000
Number of fetus	3.7±2.50	1.40±1.33	0.000
Chemical pregnancy	**10 (16.4%)**	**3 (4.8%)**	0.037
Clinical pregnancy	**10 (16.4%)**	**3 (4.8%)**	0.037

## 4. Discussion

The current study was designed and performed by the objective of comparing the treatment results in two protocols of MF and AL in treating POR patients in Intra Cytoplasmic Sperm Injection (ICSI) cycles. In the current study, the LH and Estradiol level and the number of follicles over 14 mm and endometrial thickness at the time of hCG injection were significantly higher in MF group. Besides that the mean of ovary simulation period and mean for consumption of Gonadotropin in group MF was significantly higher than AL group. These results were close to the similar studies conducted by Yarali wt al. and Davar et al. (Yarali et al., 2009, Davar et al., 2010). Meanwhile, in our study, the endometrial thickness in group MF was significantly higher than group AL, however despite in the studies of Yarali et al. and Davar et al. the endometrial thickness in group MF was higher, this difference was not statistically significant in their study (Yarali et al., 2009, Davar et al., 2010). In the current study, the number of cancelled cycles in group AL was 2.87 times more than group MF. Despite our study, in the study of Mohsen and El Din, there were no significant statistical difference in the number of oocyte and proper fetus and the number of cancelled cycles between two methods of MF and AL (Mohsen IA et al., 2013). Moreover, in the study of Yarali et al. the number of ripe oocyte were significantly higher in MF group, while in their study no significant statistical difference was observed in the number of cancelled cycles between two methods of MF and AL in poor ovarian responders (POR) (Yarali et al., 2009). In the study of Schoolcraft et al. despite the fact that the number of cancelled cycles in MF group was higher, but it didn’t have significant statistical difference with AL group. Also in their study, no significant statistical difference was observed between the two groups in the number of oocyte and proper fetus (Schoolcraft et al., 2008). In the study of Davar et al. the number of ripe oocyte was significantly higher. Also in this study, the number of cancelled cycles in AL group was higher which was not statistically significant (Davar et al., 2010). In our study, the rate of chemical and clinical pregnancies in MF method was estimated 3.85 times more than AL method. That is while Mohsen and El Din in their study have expressed similar results for these two methods (Mohsen et al., 2013). In the study of Yarali et al. despite the number of biochemical pregnancy in AL method was significantly higher, but the number of clinical pregnancy didn’t have significant statistical difference between the two methods (Yarali H et al., 2009). In the study of Davar et al. despite the number of clinical pregnancy in MF method was higher; statistically this difference was not significant in their study (Davar et al., 2010). Despite these three studies, in the study of Schoolcraft et al. similar to or study, the number of clinical pregnancy in MF method was significantly higher than AL method (Schoolcraft et al., 2008). Different studies in this field had different results and propositions in a way that study of Yarali et al. and the study of Mohsen and El Din proposed that AL method can be more helpful and economic in treating poor ovarian responders (POR), while the study of Schoolcraft et al., Davar et al. and Cakiroglu et al. similar to our study found MF method superior and more appropriate for this group of patients. It seems that more definite decision regarding the prioritized treatment method in this regard requires designing more investigations.

## 5. Conclusion

At the end, our study indicated that use of GnRH agonist flare-up protocol has more number of proper fetus, more biochemical pregnancy and the chance of more clinical pregnancy and compared to GnRH antagonist/letrozole protocol it is a more prioritized treatment for poor ovarian responders (POR).
